# Risk factors of postoperative low cardiac output syndrome in children with congenital heart disease: A systematic review and meta-analysis

**DOI:** 10.3389/fped.2022.954427

**Published:** 2023-01-10

**Authors:** Peiying Wang, Cangcang Fu, Guannan Bai, Linbo Cuan, Xiaomin Tang, Chendi Jin, Hongchong Jin, Jihua Zhu, Chunhong Xie

**Affiliations:** ^1^ Department of Pediatric Surgery, The Children's Hospital, Zhejiang University School of Medicine, National Clinical Research Center for Child Health, Hangzhou, China; ^2^ Department of Nursing, The Children's Hospital, Zhejiang University School of Medicine, National Clinical Research Center for Child Health, Hangzhou, China; ^3^ The Children's Hospital, Zhejiang University School of Medicine, National Clinical Research Center for Child Health, Hangzhou, China; ^4^ Cardiac Intensive Care Unit, The Children's Hospital, Zhejiang University School of Medicine, National Clinical Research Center for Child Health, Hangzhou, China; ^5^ Department of Cardiovascular Medicine, The Children's Hospital, Zhejiang University School of Medicine, National Clinical Research Center for Child Health, Hangzhou, China

**Keywords:** children, congenital heart disease, low cardiac output syndrome, risk factors, meta-analysis, systematic review

## Abstract

**Background:**

Low cardiac output syndrome (LCOS) is the most common complication after cardiac surgery, which is associated with the extension of postoperative hospital stay and postoperative death in children with congenital heart disease (CHD). Although there are some studies on the risk factors of LCOS in children with CHD, an unified conclusion is lack at present.

**Purposes:**

To synthesize the risk factors of LCOS after CHD in children, and to provide evidence-based insights into the early identification and early intervention of LCOS.

**Methods:**

The databases of the China National Knowledge Infrastructure (CNKI), Wanfang Database, China Science and Technology Journal Database (VIP), PubMed, Cochrane Library, Embase and Web of Science were searched for relevant articles that were published between the establishing time of each database and January 2022. Based on retrospective records or cohort studies, the influencing factors of postoperative low cardiac output in children with congenital heart disease were included in Meta analysis.This study followed the Preferred Reporting Items for Systematic Reviews and Meta-Analyses (PRISMA) guidelines. The risk of bias was evaluated according to the Newcastle-Ottawa Scale (NOS). RevMan 5.4 software was used to conduct the meta-analysis.

**Results:**

A total of 1,886 records were screened, of which 18 were included in the final review. In total, 37 risk factors were identified in the systematic review. Meta- analysis showed that age, type of CHD, cardiac reoperation, biventricular shunt before operation, CPB duration, ACC duration, postoperative residual shunt, cTn-1 level 2 h after CPB > 14 ng/ml and postoperative 24 h MR-ProADM level > 1.5 nmol/l were independent risk factors of LCOS. Additionally, the level of blood oxygen saturation before the operation was found to have no statistically significant relationship with LOCS.

**Conclusion:**

The risk factors of postoperative LCOS in children with CHD are related to disease condition, intraoperative time and postoperative related indexes, so early prevention should be aimed at high-risk children.

**Systematic Review Registration:**

https://www.crd.york.ac.uk/prospero/, identifier: CRD42022323043.

## Introduction

Congenital heart disease (CHD) is a congenital malformation caused by abnormal development of the heart and large vessels during the fetal period. At present, CHD ranks the first among birth defects in China and has become a major public health problem affecting children's physical and mental health and the quality of life (1). The report shows that there are more than 130,000 new children with CHD in China every year (2).

Low cardiac output syndrome (LCOS) is a clinical syndrome in which cardiac oxygen supply is reduced due to myocardial dysfunction and cardiovascular dysfunction, thus, insufficient oxygen can be provided to tissues and terminal organs to meet the body's metabolic needs (3). LCOS is the most common complication after cardiac surgery, which is associated with high morbidity and mortality (4).The incidence of postoperative LCOS in children with CHD is 25%∼60%, which usually occurs 6∼18 h after the operation (5–7), and the mortality rate can exceed 20% (8). The occurrence of LCOS may lead to poor prognosis, the extended hospitalization time, and the increased risk of adverse complications and high medical expenses, which brings a heavy burden on the child, family, and society (9). Therefore, reducing the incidence of postoperative LCOS in children with CHD is important to reduce the perioperative morbidity and mortality of children with CHD.

So far, there are some studies on the associated factors for the postoperative, intraoperative risk factors and postoperative risk factors. The preoperative risk factors included age, type of CHD, blood oxygen saturation, body weight, cardiac function grade and so on. The intraoperative risk factors include the duration of cardiopulmonary bypass (CPB), the type of cardioplegia, circulatory temperature and so on. The postoperative risk factors included residual shunt, 2 h cTn-1 level after CPB, 12 h ScvO2 level after CPB and so on.However, the results of studies on risk factors of LCOS in children with CHD in China and abroad are not consistence. One study (9) found that preoperative blood oxygen saturation was statistically significant with the incidence of LCOS in CHD children, which was not significant in another study (10). Mao (11) found that the preoperative left ventricular end-diastolic diameter was a protective factor for postoperative LCOS, but no other studies confirmed this conclusion.There are many similar results, and the same factor has not been uniformly confirmed in different studies.Therefore, we conducted a systematic review of the existing domestic and international publications; in addition, we applied meta-analysis to evaluate the impacts of certain risk factors on the incidence of LCOS. Efforts can be made on the modifiable factors when developing early interventions to reduce the incidence of LCOS, and eventually to improve the quality of life of children and their caregivers.

## Methods

### Search strategy

Both the systematic review and the meta-analysis were drafted according to the Preferred Reporting Items for Systematic Reviews and Meta-Analyses (PRISMA) statement. The study was registered in PROSPERO, number CRD42022323043. We searched the databases of the China National Knowledge Infrastructure (CNKI), Wanfang Database and China Science and Technology Journal Database (VIP), PubMed, Cochrane Library, Embase, and Web of Science, and the references included were searched retrospectively. The search time limit is from the establishment of the database to January 8, 2022. The following MeSH terms and free words were combined to construct systemic searches: “congenital heart disease/Heart Defects, Congenital”, “low cardiac output syndrome/Cardiac Output, Low/low cardiac output” and “risk factor*/relevant factor*/predictor/associate factors/influence*/root case analysis”.

### The diagnosis of LCOS

The diagnosis of LCOS was made if patients met more than two of following diagnostic criteria: ① Heart index < 2 L.min^−1^.m^−2^; ② Left ventricular ejection fraction < 40%; ③ Systolic blood pressure < 90 mmHg or systolic blood pressure decreased by more than 20% compared with preoperative blood pressure; ④ Central venous pressure > 15 cm H2O, or prolonged capillary refill > 3s or Central venous oxygen saturation < 50%; ⑤ Postoperative dopamine dosage > 10ug/ (kg.min) can maintain systolic blood pressure and cardiac output, and the duration of administration is longer than that of 30 min; ⑥ Lactic acid > 3.5 mmol/L, or metabolic acidosis(PH < 7.4, Lactic acid > 3.0 mmol/L, base excess < −2 mmol/L); ⑦ Urine volume < 0.5 ml/(kg.h) for more than 2 h; ⑧ The difference between the central temperature and the peripheral temperature > 5°C, and the limbs were cold.

### Types of populations

The subjects were postoperative children with CHD (age<18 years old).

### Types of interventions and comparators

According to whether LCOS occurred after operation, the patients were divided into LCOS group and no LCOS group.

### Types of outcome measures

The risk factors of postoperative LCOS in children with CHD were obtained by multivariate regression analysis.

### Types of studies

The type of study was prospective study or retrospective, and the published language was English or Chinese. The data of odd ratio (OR), 95% confidence interval (CI), and standard error (SE) or the values of the mean (x) and standard deviation (s) were provided or converted into the results of the study.

### Exclusion criteria

Incomplete or contradictory data, publications without peer review and repeated publication or without full text are excluded from review.

### Data extraction

The data extraction was carried out independently by two researchers (WPY and CLB) according to the criteria of literature inclusion and exclusion. If there was any disagreement, it would be solved by discussing it with each other or consulting the third researcher. The contents of the data extraction included author, country, study period, participants, type of study, sample size, age, and findings.

### Quality assessment

The quality was evaluated independently by two researchers (WPY and CLB) using the Newcastle-Ottawa scale (NOS), and the evaluation results were checked. If they disagreed, they were resolved by discussing with each other or consulting a third researcher. NOS included the selection of subjects (4 items, 1 point each), comparability between groups (1 item, 2 points), and exposure or outcome evaluation (3 items, 1 point each), with a total score of 9. The score ≥ 7indicates a good quality, while < 7 indicates a poor quality.

### Meta-analysis

RevMan 5.4 software was used for statistical analysis. The statistical effects of counting data were expressed by OR and 95%CI, while those of continuous data were expressed by mean difference (MD) and 95%CI. If *p* < 0.1 and *I*^2^ ≥ 50%, it suggests that a heterogeneity exists between studies. Sensitivity analysis was used to explore the source of heterogeneity, and the comprehensive effect was calculated after excluding heterogeneity. A fixed-effect was selected for meta-analysis.

## Results

### Study selection

**Figure 1:**
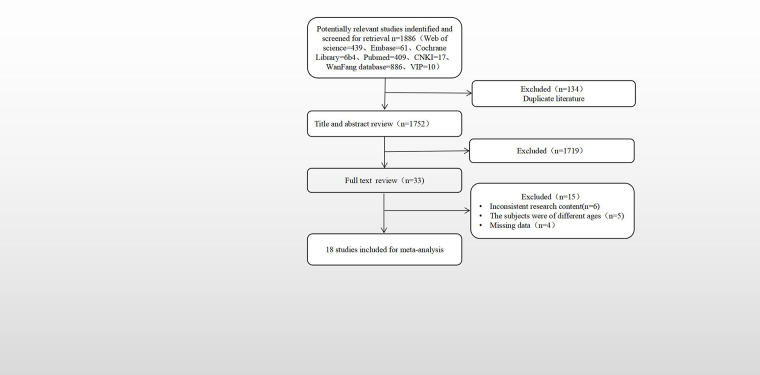
Flow diagram for the selection of included articles.

A total of 1,886 articles were searched and selected, including 913 in Chinese and 973 in English. After the repetitive literature was excluded, there were 1,752 reading titles and abstracts, and 1,719 reading titles and abstracts were deleted. The remaining 33 read the full text, 15 were excluded after reading the full text, and finally included 18,9 in Chinese and 9 in English. The literature screening process and results are shown in Figure 1.

### Characteristics of included studies

Table 1 presents the general characteristics and main findings of the included studies. Among the 18 articles, there were 11 prospective studies and 7 retrospective studies,. The total sample size was 12,048, and the LCOS case group size was 1,681. The incidence of LCOS was 13.95%. The publication time of the study is from 2007 to 2021.

**Table 1:** Characteristics of the included studies.

	Authors	Year	Country	Study period	Participants	Type of the study design	Sample size (number of LCOS cases)	Age ($\bar{x}\pm s$) (Days OR Years OR Month)		Main findings
								LCOS	No LCOS	
1	Song et al. (9)	2021	China	2019.01- 2020.10	Children (<14) with CHD.	Retrospective	283 (35)	3.1 ± 2.6 (Y)	6.9 ± 5.2 (Y)	Age ≤ 4y, preoperative oxygen saturation ≤93%, biventricular shunt before operation, duration of CPB ≥ 60 min,the postoperative residual shunt was the independent risk factor of LCOS in patients with CHD.
2	Drennan et al. (12)	2021	USA	2017.06-2018.12	Children with CHD born ≥36 weeks gestational age, with a birth weight greater than 2.5 kg, and less than 6 months old.	Prospective	26 (11)	20.2 ± 22.5 (D)	21.4 ± 34.0 (D)	The duration of aortic cross clam (ACC) and, the level of postoperative IL-8 were independent risk factors of LCOS in patients with CHD.
3	Iliopoulos et al. (10)	2020	USA	2015.07	Children with CHD (<17 were admitted to ICU after cardiac surgery.	Prospective	47 (6)	NA	NA	In children after congenital heart surgery, their preoperative neutrophil-lymphocyte ratio was associated with a higher chance of low cardiac output in the postoperative period.
4	Du et al. (11)	2020	China	2014.01-2017.12	Children with CHD (<18 y) after cardiac surgery.	Retrospective	8,660 (864)	272.5 ± 307.1 (D)	501.4 ± 569.8 (D)	Age, tricuspid regurgitation, Risk Adjustment in Congenital Heart Surgery-1 risk grade, aortic shunt, atrial shunt, ventricular level shunt, postoperative residual shunt, left ventricular outflow tract obstruction, right ventricular outflow tract obstruction, circulating temperature, duration of CPB, myocardial preservation using histidine-tryptophan-ketoglutarate, and mitral insufficiency were independent risk predictors of LCOS in patients with CHD.
5	Xiang et al. (13)	2020	China	2012.01-2018.12	Children with CHD (≤14y)who underwent correction of intracardiac malformation under CPB.	Retrospective	476 (45)	3.1 ± 4.3 (Y)	6.9 ± 6.2 (Y)	Age, biventricular shunt before operation and cardiac reoperation, ACC duration, and postoperative residual shunt were independent risk factors for LCOS after CHD in children.
6	Mao et al. (14)	2020	China	2014.01-2018.01	Children with CHD (<18 y) need total anomalous pulmonary venous connection.	Retrospective	153 (50)	3.00 ± 3.1 (M)	6.41 ± 7.5 (M)	The preoperative left ventricular end-diastolic diameter and preoperative oxygen saturation were protective factors for LCOS after TAPVC, while the CPB duration was an independent risk predictor of LCOS.
7	Dai et al. (15)	2020	China	2017.04-2018.03	Children with CHD (<18 y) were admitted to PICU after CPB.	Prospective	70 (22)	52.56 ± 26.9 (M)	79.78 ± 39.7 (M)	The level of cTn-1 at 2 h after CPB and the oxygen saturation at 12 h after CPB were independent risk factors of LCOS.
8	Murni et al. (16)	2019	Canada	2014.04-2015.03	Children with CHD (<18 y) were admitted to CICU after CPB.	Prospective	257 (51)	NA	NA	Predictors of LCOS were cyanotic CHD, longer duration of CPB, high inotropes, and an increase in lactate >0.75 mmol/l/h or more in the first 24 h.
9	Perez-Naveroet al. (17)	2019	Spain	NA	Children with CHD (<18 y) were admitted to PICU after CPB.	Prospective	115 (33)	NA	NA	Age, duration of CPB, VIS score, The level of cTn-1 at 2 h after CPB, and the level of MR-ProADM at 24 h after CPB were independent risk predictors of LCOS.
10	Sobieraj et al. (18)	2018	Poland	2006-2012	Children with CHD (<18 y) after CPB.	Retrospective	1,129 (399)	NA	NA	Age, duration of CPB, presence of specific CHDs, cardiac reoperation, the urgency of operation, operation time, and crystalloid cardioplegia were independent risk predictors of LCOS.
11	Pérez-Navarro et al. (19)	2017	Spain	NA	Children with CHD (<18 y) were admitted to PICU after CPB.	Prospective	117 (33)	NA	NA	The level of cTn-1 at 2 h after CPB and the level of MR-ProADM at 24 h after CPB were independent risk predictors of LCOS.
12	Wang et al. (20)	2014	China	2011.01-2014.07	Children (<18 y) with CCHD.	Prospective	60 (15)	5.6 ± 2.1 (M)	7.5 ± 2.3 (M)	Age, duration of CPB, BNP before the operation, and BNP 6 h after the operation are independent predictors of LCOS.
13	Zhou et al. (21)	2011	China	2001.01-2010.12	Children with (<18 y) Tetralogy of Fallot.	Prospective	191 (20)	6.75 ± 1.3 (M)	14.35 ± 4.6 (M)	Age ≤ 6 months, Nakata index < 140mm^2^/m^2^, perioperative accident, and the duration of CPB > 150 min were the risk factors of LCOS after radical resection of TOF in children.
14	Yang (22)	2009	China	2008.05-2008.07	Children with CHD (<18 y) after CPB.	Prospective	22 (5)	NA	NA	The level of NT-proBNP before CPB was an independent risk predictor of LCOS.
15	Wang et al. (23)	2008	China	2004.01-2007.12	Children with CHD (<18 y) after CPB.	Retrospective	310 (21)	NA	NA	Age, the duration of CPB, type of CHD, and cardiac function before CPB were independent factors of LCOS.
16	Song et al. (24)	2008	China	NA	Children with complex CHD (<18 y) undergoing radical surgery	Prospective	64 (30)	9.8 ± 11.3 (M)	18.3 ± 21.5 (M)	Age, body weight, preoperative pulmonary hypertension, risk Adjustment for Congenital Heart Surgery, ACC duration, CPB duration, and high systemic and pulmonary vascular resistance after operation were the risk of LCOS.
17	Carmona et al. (25)	2008	USA	2017.06-2018.11	Infants younger than 6 months with CHD.	Prospective	46 (29)	7.23 ± 14.0 (M)	8.78 ± 12.9 (M)	The level of NT-proBNP before CPB and the level of postoperative IL-8 were independent risk predictors of LCOS.
18	Zhu et al. (26)	2007	China	2000.01-2005.12	Low birth weight children with CHD.	Retrospective	22 (12)	NA	NA	NA

### Quality assessment

Table 2 shows the results of the methodological quality evaluation. The scores of quality evaluation of 18 studies were all above 7 points.

**Table 2:** Included study quality evaluation.

Study	Quality evaluation (points)			NOS total score
	Selection	Selection of the non-exposed cohort	Exposure or outcome	
Song (2021)	4	2	2	8
Drennan (2021)	4	1	3	8
Iliopoulos (2020)	3	2	2	7
Du (2020)	4	2	2	8
Xiang (2020)	4	2	2	8
Mao (2020)	4	2	2	8
Dai (2020)	4	2	3	9
Murni (2019)	4	1	2	7
Perez-Navero (2019)	4	2	3	9
Sobieraj (2018)	4	1	2	7
Pérez-Navero (2017)	4	2	3	9
Wang (2014)	4	1	2	7
Zhou (2011)	4	2	1	7
Yang (2009)	4	2	3	9
Wang (2008)	3	1	3	7
Song (2008)	4	1	3	8
Carmona (2008)	4	2	3	9
Zhu (2007)	3	1	2	7

### Meta- analyses

**Figure 2:**
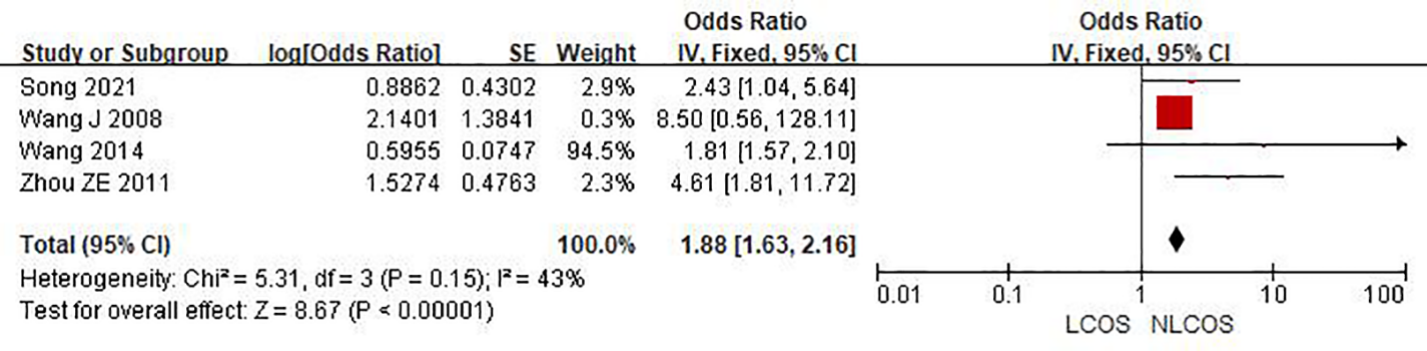
Age of children in forest plot.

**Figure 3:**
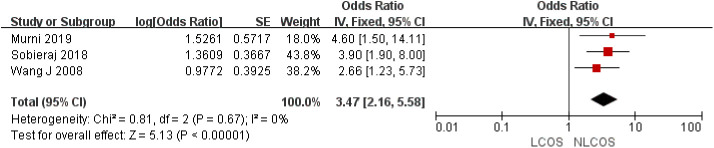
Types of congenital heart disease forest plot.

**Figure 4:**
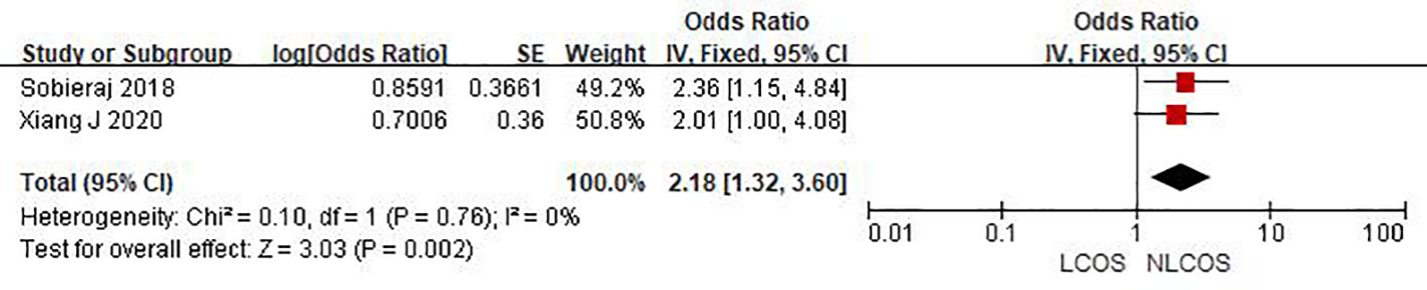
History of a cardiac surgery forest plot.

**Figure 5:**
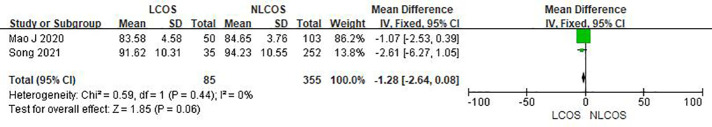
Preoperative oxygen saturation forest plot.

**Figure 6:**
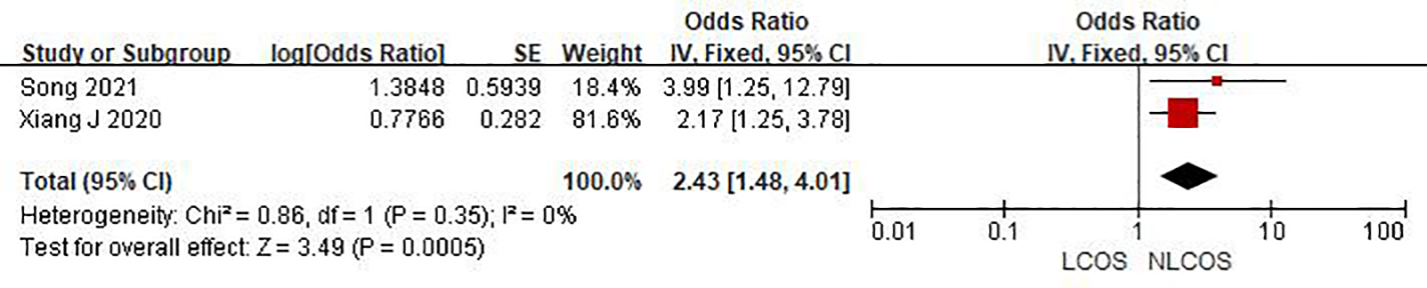
Biventricular shunt before operation forest plot.

**Figure 7:**
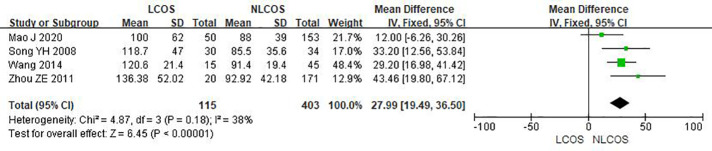
Duration of CPB forest plot.

**Figure 8:**
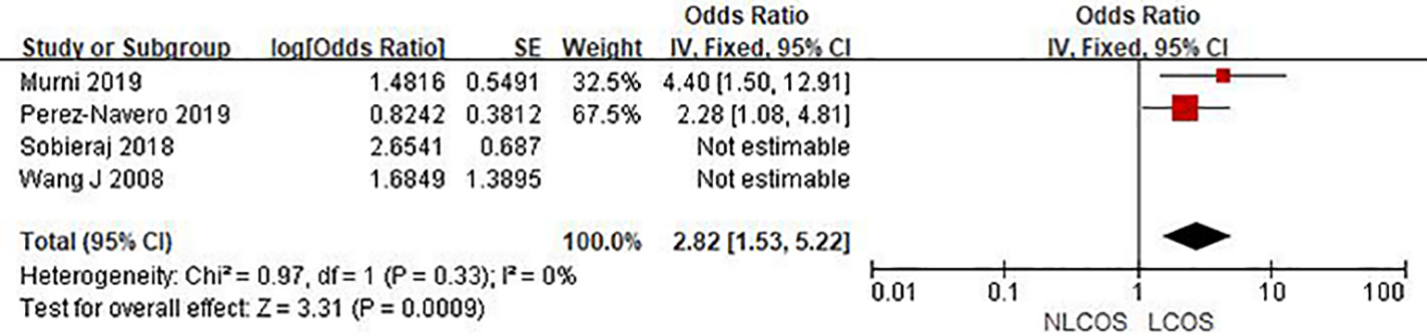
CPB duration forest plot.

**Figure 9:**
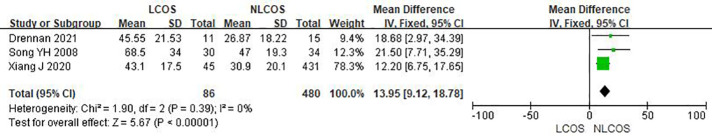
ACC duration forest plot.

**Figure 10:**
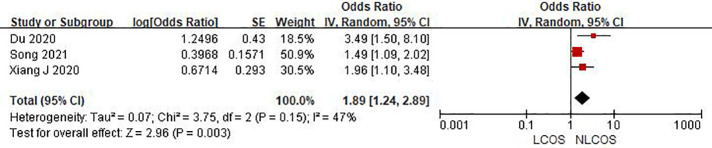
Postoperative residual shunt forest plot.

**Figure 11:**
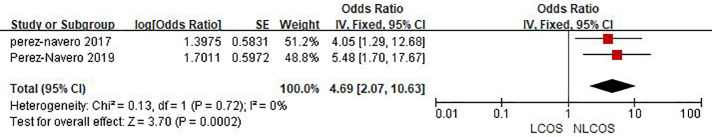
CTn-1 level 2 hours after CPB forest plot.

**Figure 12:**
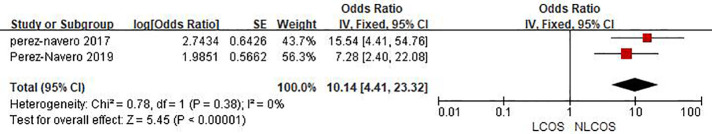
Postoperative 24h MR-ProADM level forest plot.

Nine studies reported that the age of children was related to postoperative LCOS in children with CHD, among them, seven studies had the same data type, and meta analysis showed that there was heterogeneity between the results (I^2^: 93%, *P* < 0.0001). Sensitivity analysis, 3 articles causing heterogeneity were excluded, and the remaining 4 articles did not have heterogeneity (OR = 1.88, 95% CI: 1.63,2.16; *P* < 0.001, Figure 2); Three studies reported the effect of the type of CHD on the incidence of LCOS, and there was no heterogeneity between the results (OR = 3.47, 95% CI:2.16,5.58; *P* < 0.001; Figure 3); Two studies reported that re-cardiac surgery was associated with postoperative LCOS of CHD (OR = 2.18, 95% CI:1.32,3.60; *P* = 0.002, Figure 4); Two studies reported that preoperative blood oxygen saturation was associated with postoperative LCOS of CHD, and there was no heterogeneity between the results. However, the level of blood oxygen saturation before the operation were not significant (OR = −1.28, 95% CI:-2.64,0.08; *P* = 0.06, Figure 5); Two studies reported that the presence of biventricular shunt before operation was associated with postoperative LCOS of CHD (OR = 2.43, 95% CI:1.48,4.01; *P* = 0.0005, Figure 6); Ten studies reported that CPB duration was related to postoperative LCOS in children with CHD. Among them, six studies had the same data type and meta analysis showed that there was heterogeneity between the results (I^2^: 83%, *P* < 0.0001). Sensitivity analysis, 2 articles causing heterogeneity were excluded, and the remaining 4 articles did not have heterogeneity. Fixed effect model was used for analysis. It was concluded that CPB duration was the influencing factor of postoperative LCOS in children with CHD (MD = 27.99, 95% CI:19.49,36.50; *P* = 0.00001, Figure 7). The results of the other two studies reported the relationship between the duration of CPB > 120 min and postoperative LCOS in children with CHD, using fixed effect model for combined analysis, it was concluded that the duration of CPB > 120 min was the influencing factor of postoperative LCOS in children with CHD (OR = 2.82, 95% CI:1.53,5,22; *P* = 0.0009, Figure 8);Three studies reported the effect of ACC duration on the occurrence of LCOS, and there was no heterogeneity between the results (MD = 13.95, 95% CI:9.12,18.78; *P* < 0.00001, Figure 9); Three studies reported the effect of postoperative residual shunt on the occurrence of LCOS, and there was no heterogeneity among the results. Using fixed effect model for combined analysis, it was concluded that postoperative residual shunt was a risk factor for postoperative LCOS of CHD (OR = 1.89, 95% CI:1.24,2.89; *P* < 0.001, Figure 10); Two studies reported the correlation between the level of 2 h cTn-1 > 14 ng/ml after CPB and the occurrence of LCOS after CHD (OR = 4.69 95% CI:2.07,10.63; *P* < 0.001, Figure 11); Two studies reported that postoperative 24 h MR-ProADM > 1.5 nmol/L was associated with postoperative LCOS in CHD (OR = 10.14, 95% CI:4.41,23.32; *P* < 0.001, Figure 12).

## Discussion

This review collected 18 studies that reported the risk factors of LCOS after cardiac surgery and identified nine risk factors including age, type of CHD, cardiac reoperation, biventricular shunt before operation, CPB duration, ACC duration, postoperative residual shunt, cTn-1 level 2 h after CPB > 14 ng/ml and postoperative 24 h MR-ProADM level > 1.5 nmol/L.

A total of nine studies reported that age was related to postoperative LCOS in children with CHD. Four articles reported that age was related to postoperative LCOS in children with CHD without heterogeneity were analyzed by fixed effect model, and it was concluded that age was the influencing factor of postoperative LCOS in children with CHD. The results of the included articles all showed that younger children with CHD had a higher risk of developing LCOS. However, according to the results of the study, we are unable to determine the specific age of the child is more useful for clinical early warning. Moreover, few studies have stratified the age of the children, and the age nodes of the stratification are different, some are four years old (9), some are six months (21). More studies are needed to explore the relationship between different age stratification and postoperative LCOS in children with congenital heart disease.

Re-cardiac surgery mainly occurs in complex intracardiac malformations that need to be corrected by stages and residual or secondary lesions need to be further corrected. Jacobs (27) according to the data of the Society of Thoracic Surgeons Congenital Heart Database (STSCHD database), the operation situation of CHD children in North America from 2007 to 2011 was about 1/3 for re-operation, and the incidence of LCOS is significantly increased after re-operation.

We found that the longer the CPB duration, the higher the risk of postoperative LCOS in children with CHD, but which node the CPB duration exceeds has more early warning effect on the clinic. This study can not draw a conclusion. four of the included studies divided CPB duration nodes, two studies (16) were divided by CPB duration > 120 min, and one study (9) was divided by CPB duration > 60 min. Another item (23) divides the CPB duration into < 50 min, 50∼100 min, 100∼150 min, and > 150 min. More research is needed to find the duration of CPB that can be used as an early warning for clinical practice.

During aortic occlusion, the heart is in a state of inhibition, ischemia, and hypoxia (28). After the opening of the aorta, myocardial reperfusion induces systemic inflammation and endothelial cell activation, resulting in intracellular oxygen free radicals and calcium overload, resulting in cardiomyocyte injury and affecting the systolic and diastolic function of the heart (29). Drennan (12) found that children with ACC duration > 45 min have a higher risk of developing LCOS after operation, but the sample size of this study is smaller, and larger sample size studies are needed to prove this point.

The postoperative residual shunt is a common postoperative complication in children with CHD, with an incidence of 5∼25% (30, 31). Postoperative residual shunt mainly occurred in children with intracardiac malformations with severe pulmonary hypertension (17), most of these children were in a serious condition and had a poor basic cardiac function (32). Hemodynamic abnormalities caused by residual shunts can also aggravate the myocardial injury and eventually lead to LCOS.

The level of cTn-I increases rapidly after cardiac injury, which could be used as a predictor of cardiac biomarkers in patients with coronary heart disease after cardiac surgery (33). Bojan (34) describe that early increases in cTn-I levels may help predict the course of disease in newborns and infants after heart surgery. Many studies have shown that MR-proADM alone or in combination with other indicators can predict LCOS in children with CPB (35, 36).

At present, it has been agreed that LCOS is a risk factor for poor prognosis after cardiac surgery (37). Through strict monitoring of cardiac output indicators, early diagnosis of LCOS and early identification the causes can help to reduce mortality, and improve the prognosis. Therefore, the timing of the operation should be strictly grasped according to the disease and age of the children. When physicians communicate with family members whose children are at high risk of LCOS, the risk factors should be emphasized to help family members adjust their expectations on the operation and prognosis, which may avoid the miscommunication between professionals and patients as well as their caregivers. Operation should be carefully conducted to reduce the residual shunt and avoide a second operation. Physicians should constantly improve their surgical techniques to shorten the aortic cross-clamping time as much as possible, and to improve the perioperative management and identification of LCOS.

### Strengthes and limitations

This systematic review includes not only articles published in English, but also articles published in Chinese as many as possible. To our best knowledge, it has covered all the relevent studies on the risk factors of postoperative LCOS of CHD that we could found in both domestic and international databases. In addition, the meta-analysis of this study used sensitivity analysis to explore the sources of heterogeneity on the basis of heterogeneity test, and calculated the comprehensive effects after excluding the heterogeneity study, which increased the reliability of results.

However, there are still several limitations warranting for attention. First, there are great differences in the sample size and age distribution of patients in each study, which may lead to the dispersion and heterogeneity of the included survey results. Second, there are many scattered influencing factors included in the literature report, On the other hand, there are few articles on some influencing factors, so it is suggested that a large sample multicenter study should be carried out in the future to further explore the incidence and risk factors of postoperative LCOS in patients with congenital heart disease.

## Conclusion

A total of 1,886 records were screened, of which 18 were included in the final review. We have identified 9 risk factors were found in the systematic review. Meta-analysis demonstrated that age, type of CHD, reoperation, biventricular shunt before the operation, CPB time, ACC time, postoperative residual shunt, cTn-1 level 2 h after CPB > 14 ng/ml and postoperative 24 h MR-ProADM level > 1.5 nmol/l were independent risk factors of LCOS. Efforts can be made on the modifiable factors when developing early interventions to reduce the incidence of LCOS, and eventually to improve the quality of life of children and their caregivers.

## Data Availability

The original contributions presented in the study are included in the article/Supplementary Material, further inquiries can be directed to the corresponding author/s.
